# Ultra-Short-Term Dual Antiplatelet Therapy in Treating Unruptured Brain Aneurysm With the Pipeline Flex-Shield Embolization Device

**DOI:** 10.7759/cureus.25516

**Published:** 2022-05-31

**Authors:** Adam Delora, Obadah Ezzeldin, Uzma Ali, Mohammad El-Ghanem, Mohamad Ezzeldin

**Affiliations:** 1 Emergency Medicine, HCA Houston Kingwood/University of Houston College of Medicine, Kingwood, USA; 2 Radiology, University of Texas Medical Branch, Galveston, USA; 3 Neurology, HCA Houston Kingwood/University of Houston College of Medicine, Kingwood, USA; 4 Neuroendovascular Surgery, HCA Houston Northwest/University of Houston College of Medicine, Houston, USA; 5 Neuroendovascular Surgery, HCA Houston Kingwood/University of Houston College of Medicine, Kingwood, USA

**Keywords:** internal carotid, pipeline flex, cerebral aneurysm, sapt, dapt, flow diverter, intracranial aneurysm, pipeline shield

## Abstract

Ruptured cerebral aneurysms can cause significant morbidity and mortality. Endoluminal devices to treat aneurysms such as the Pipeline™ Flex Embolization Device with Shield Technology (PFES) (Medtronic, Dublin, Ireland) integrate phosphorylcholine on the surface of the device in order to reduce platelet adherence that causes periprocedural thromboembolic events and subsequent long-term intrastent stenosis. In addition to the Shield Technology, patients are commonly placed on dual antiplatelet therapy (DAPT) for six months to reduce thromboembolic events and subsequent long-term intrastent stenosis. There is a strong positive correlation between the length of DAPT use and bleeding. Here, we present a case of a 66-year-old female with a right supraclinoid internal carotid artery (ICA) aneurysm treated with a PFES who was placed on dual antiplatelet therapy for the first 31 days postoperative and subsequently maintained on aspirin (ASA) 81 mg monotherapy. At two months, a follow-up diagnostic cerebral angiogram showed complete occlusion of the aneurysm with a patent stent. Our case sets the stage for further research into the optimal length of dual antiplatelet therapy required in PFES to prevent short and long-term thromboembolic events. This report indicates that it may be safe for patients with PFES to intermittently halt the use of DAPT to manage bleeding complications or perform surgery.

## Introduction

Over the years, there have been several incremental advances in the endovascular treatment of intracranial aneurysms [1]. Flow diversion devices divert flow into the parent vessel and disrupt the flow of blood into the aneurysm resulting in aneurysm thrombosis and eventually epithelialization at the neck of the aneurysm. Over time epithelialization over the stent will result in occlusion of the aneurysm [2]. A few of the manufacturers and their device lines include Medtronic (Pipeline; Dublin, Ireland), Stryker (Surpass; Kalamazoo, Michigan), MicroVention (FRED; Aliso Viejo, California), and Balt Extrusion (Silk; Montmorency, France) [3]. 

The most common type of complication associated with aneurysm treatment using flow diverters, coils, and stent-assisted coiling is thromboembolic complications [4-7]. Medtronic Neurovascular introduced a surface modification to the Pipeline Flow device named Shield Technology. Shield Technology reduces perioperative thromboembolic events associated with flow diverting devices [7]. Shield Technology integrates phosphorylcholine on the surface of the device to reduce platelet adherence that causes periprocedural thromboembolic events and subsequent long-term intrastent stenosis. Phosphorylcholine is found on the surface of red blood cells. A synthetic type of phosphorylcholine polymer is bonded to the braid of the Pipeline device. In-vitro, ex-vivo, and in-vivo studies have shown decreased thrombogenicity of devices with Shield Technology [6-12].

In order to reduce the incidence of thrombotic complications, patients are also placed on antiplatelet therapy with one or two medications called single antiplatelet therapy (SAPT) and dual antiplatelet therapy (DAPT), respectively. However, the benefits associated with DAPT are counterbalanced by a higher bleeding risk correlated to the treatment duration [13]. In a porcine model, the Pipeline Flex Embolization Device with Shield Technology (PFES) showed faster endothelial growth and comparable neointimal volume than the same device without the Shield Technology when using single antiplatelet therapy of ASA at 10 mg/kg. Also of note, PFES with SAPT had significantly less thrombus on the vascular surface than PED-Flex without Shield Technology. However, PFES with SAPT did demonstrate a higher rate of thrombus compared with PED-Flex under DAPT [12]. In a one-year prospective trial performed post-market, 87.7% of subjects interrupted DAPT, but 75.7% (109/144) had aneurysm occlusion with a patent parent vessel that did not require retreatment [14]. In a systematic review of flow diverters, few had available data on DAPT duration. In those studies that included the data, DAPT was generally prescribed for at least four months but six was more common. Standard regimens included acetylsalicylic acid (ASA) 75-325 mg along with a second antiplatelet agent. Common agents included clopidogrel 75-150 mg daily and ticagrelor 90 mg twice daily. After DAPT was completed, this was followed by variable doses of ASA for at least six months to indefinitely [15]. Although long-term data was not present in this publication, a small-scale study (14 patients) evaluated PFES in patients with aneurysmal subarachnoid hemorrhage (SAH) receiving SAPT. At early follow-up at and around Day 7, there was complete or near-complete aneurysm occlusion in 12 (85.7%) patients [16]. In a case report, PFES was used with ASA monotherapy in a ruptured fusiform vertebral artery aneurysm and had stent thrombosis on Day 10 [17]. All of this taken together indicates that DAPT is likely necessary, but the standard six months may not be optimal.

The optimal duration of DAPT in patients with PFES devices is unclear. Here, we present a 66-year-old female with an unruptured brain aneurysm, treated with a PFES device. She received DAPT for 31 days and then subsequently only received ASA 81 mg daily. A subsequent angiogram showed the device occluding the aneurysm without evidence of stenosis of the parent vessel. To our knowledge, we present the first case of a patient with an unruptured aneurysm treated with a Pipeline Flex Embolization Device with Shield Technology who received an ultra-short course of DAPT.

## Case presentation

**Figure 1:**
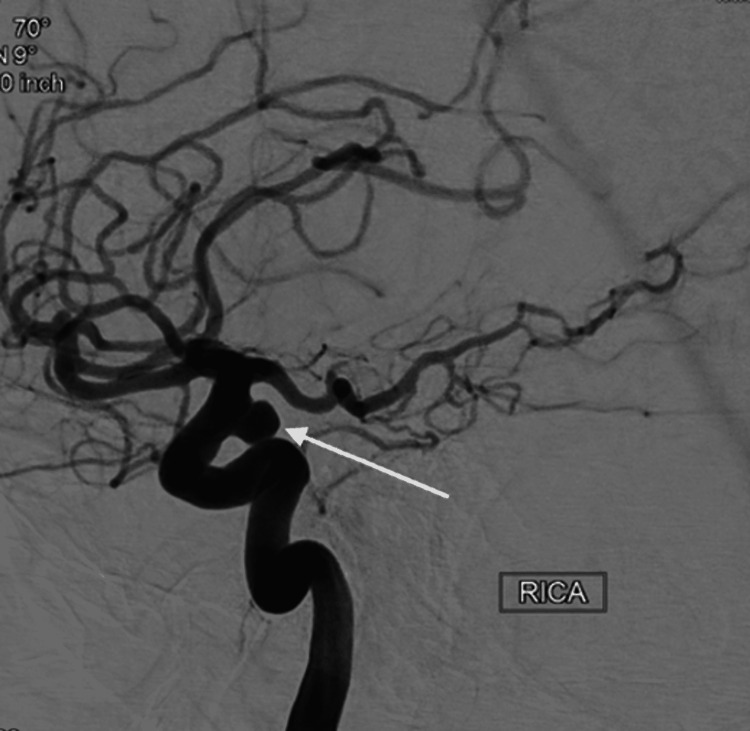
Right ICA angiogram, lateral view demonstrating a right supraclinoid internal carotid artery aneurysm This is a lateral view of a conventional cerebral angiogram. The contrast was injected into the right internal carotid artery (ICA). The white arrow points to the aneurysm.

A 66-year-old right-handed female, with a past medical history significant for hyperlipidemia, hypertension, prior stroke without residual deficits, chronic neck pain, neuropathy, and restless leg syndrome, a former smoker for 30 years, was found to have an incidental finding of a supraclinoid right internal carotid artery (ICA) aneurysm on CT Angiography during a workup for acute left basal ganglia stroke resulted in right hemibody weakness. This finding was confirmed three months later on diagnostic angiography, showing a right internal carotid artery aneurysm measuring approximately 6.94 x 4.5 x 5.19 mm and a neck of 3.74 mm as demonstrated in Figure 1.

**Figure 2:**
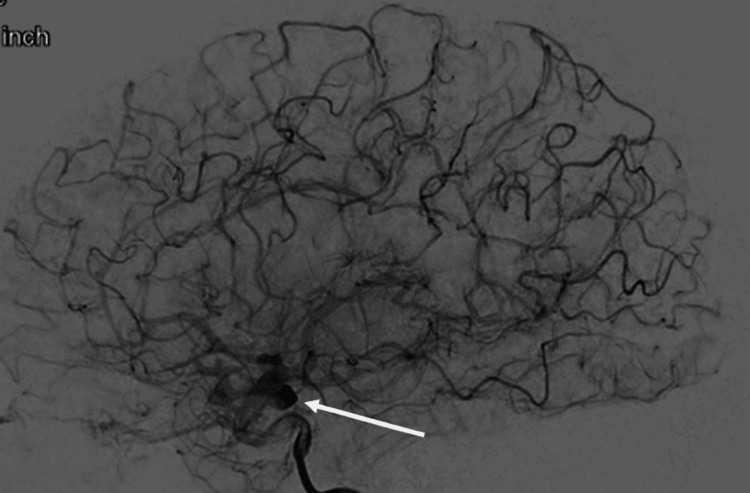
Right ICA angiogram, lateral view, demonstrating immediate contrast stagnation post-aneurysm treatment with a pipeline embolization device This is the lateral view of a conventional cerebral angiogram. The contrast was injected into the right internal carotid artery (ICA). The white arrow points to the aneurysm. This was taken immediately after the Pipeline™ Flex Embolization Device with Shield Technology (PFES) was placed. It shows contrast stagnation. The PFES disrupts the normal blood flow into the aneurysm. This disrupted blood flow is represented by the contrast stagnation into the aneurysm. This device is designed to cause thrombosis in the aneurysm and eventually occlusion of the aneurysm.

On Day 0, a 5 x 14 mm Pipeline Flex Embolization Device with Shield Technology was placed, resulting in significant and immediate contrast stagnation, as shown in Figure 2. The device appeared to have good wall apposition and no evidence of endoleak or in-stent stenosis. Her neurological exam was unchanged from admission. She was discharged the following day with instructions to take DAPT of ASA 325 mg and clopidogrel 75 mg daily.

On follow-up on postop Day 25, the patient complained of right groin pain and was found to have a moderate-sized superficial leg hematoma that resulted in local tenderness. Platelet reactivity test to Plavix (PRP) was found to be 5 P2Y12 Reaction Units (PRU). The normal PRP range is 194-418 PRU with values below 194 PRU to have specific evidence of a P2Y12 inhibitor effect. The clopidogrel could not be titrated down further so it was stopped and ticagrelor was started. The DAPT regimen was titrated down to ASA 81 mg and ticagrelor 30 mg BID with a follow-up PRP of 23 and 46 PRU. Medication compliance was emphasized.

**Figure 3:**
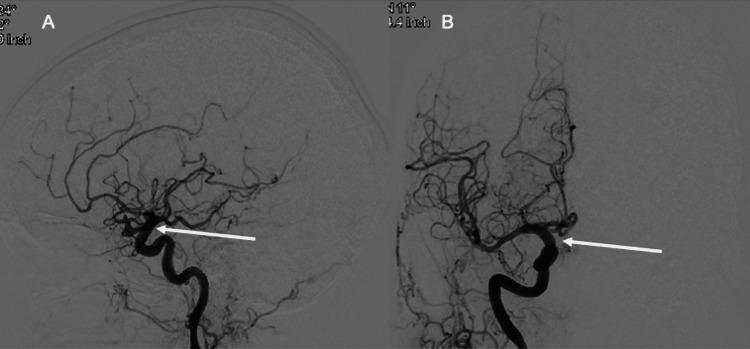
Follow-up right common carotid artery angiogram, lateral view (A) and AP view (B), demonstrating no residual filling of the aneurysm, a patent stent, and good wall apposition This is a conventional cerebral angiogram. The contrast was injected into the right common carotid artery. Panel A shows a lateral view and panel B shows an anterior-posterior (AP) view. The white arrow in both panels points to the site of the original aneurysm. There does not appear to be any residual filling of the aneurysm. The stent is patent.

On Day 62, the patient revealed she was not taking the ticagrelor and was only on ASA 81 mg daily. Same day PRP was found to be 239 PRU. As shown in Figure 3, the conventional cerebral angiogram demonstrated no residual filling of the aneurysm, Raymond-Roy occlusion class I. The PFES appeared patent with good wall apposition and no evidence of in-stent stenosis. There was normal antegrade flow in the right ICA and its distal branches. No neurological sequelae were appreciated on a physical exam from this ultra-short-term DAPT regimen. The patient was restarted on the DAPT regimen of ASA 81 mg and ticagrelor 30mg BID.

## Discussion

It has been estimated that 2-3% of the population of the world has an unruptured intracranial saccular aneurysm [18]. The incidence of aneurysmal SAH is estimated to be 7.9 per 100,000 person-years. Roughly one-third of patients with ruptured aneurysmal SAH die in the first days to weeks and in those that survive, there is significant morbidity [19]. Historically, aneurysms were treated with invasive techniques like removing a bone flap and placing a clip on the aneurysm to obstruct the flow of blood into the aneurysm. Since then, a number of new techniques have been developed. Endovascular coiling can be performed. In this procedure, detachable wires are placed into the aneurysm obstructing the flow of blood. This causes a clot and eventually endothelialization over the neck of the aneurysm. Another endovascular treatment modality includes flow diverters, which divert a portion of the blood away from the aneurysm, causing thrombosis in the aneurysm. Epithelialization occurs over the aneurysm. The two types of flow diverters are intrasaccular flow diverters that sit in the aneurysm sac and intravascular flow diverters that sit in the parent vessel. One intrasaccular device is the Woven EndoBridge (WEB), which is made by Sequent Medical (Aliso Viejo, California). The original intravascular flow diverter was approved by the FDA in 2011 by Covidien, which merged with Medtronic. This device was called the Pipeline Embolization Device (PED). It has gone through two evolutions. The Pipeline Flex Embolization Device was released that employed a new delivery system. The SHIELD trial was performed to evaluate the safety and efficacy of the Pipeline Flex Embolization Device with Shield Technology. On March 25, 2016, the trial began and the first results were posted on July 8, 2020 [2,14].

The biomembrane mimicry of cell membranes using phosphorylcholine, which is used in the PFES device, reduces intrastent stenosis by reducing platelet adhesion and activation [8]. In both the rabbit and porcine models, PFES showed reduced thrombus formation compared to devices without Shield Technology. In the porcine model, the PFES also showed increased endothelialization. The PFES with SAPT did demonstrate a slightly higher rate of thrombus compared with PED-Flex without Shield Technology with DAPT [11-12]. In a baboon ex-vivo model that looked at a Pipeline Flow Embolization Device with and without Shield technology and Flow-Redirection Endoluminal Device (FRED), PFES showed less fibrin and platelet deposition when using SAPT [10]. Therefore, there is experimental evidence to support the possibility that using a shorter duration of DAPT in patients with a PFES than the previously cited six months may be optimal but only using SAPT may increase the risk of stenosis. In fact, in one case report with a patient with subarachnoid hemorrhage and active malignancy, the PFES was used with ASA monotherapy in a ruptured fusiform vertebral artery aneurysm and had stent thrombosis on day 10 [17]. Also, there was a case report where a patient had in-stent thrombosis on Day 13 following PFES placement on SAPT with ASA 81 mg [20]. The benefits associated with DAPT are counterbalanced by a higher bleeding risk correlated to the treatment duration [13].

The current standard of care for Pipeline Flex Embolization Devices with and without Shield Technology is six months [14-15]. We report a patient who had a Pipeline Flex Embolization Device with Shield Technology who only underwent 31 days of DAPT followed by ASA 81 mg and had no evidence of stenosis. This case and the existing literature suggest that a shorter duration of DAPT in patients who had a stent placed utilizing Shield Technology may be used. Future research should determine the optimal duration of DAPT following placement of Pipeline Flex Embolization Devices with Shield Technology. This patient was on DAPT for 31 days and then only on ASA 81 mg daily for 30 days without thromboembolic complications and no residual filling of the aneurysm. This indicates that a shorter DAPT therapy duration might be safely considered, especially in situations such as bleeding or emergency surgery.

## Conclusions

To our knowledge, we present the first case of a patient with an unruptured aneurysm treated with PFES devices who received an ultra-short course of DAPT. In addition to the existing literature, this case report indicates that larger-scale studies should be performed in determining the optimal length of treatment with dual antiplatelet therapy in patients with flow diverters utilizing shield technology. Larger studies could potentially radically reduce the duration of DAPT use in patients with PFES devices and determine the risk of in-stent stenosis in shorter courses of DAPT.
